# Thyroid Hormone Transporters in Pregnancy and Fetal Development

**DOI:** 10.3390/ijms232315113

**Published:** 2022-12-01

**Authors:** Zhongli Chen, Marcel E. Meima, Robin P. Peeters, W. Edward Visser

**Affiliations:** Academic Centre for Thyroid Diseases, Department of Internal Medicine, Erasmus University Medical Center Rotterdam, 3015 GD Rotterdam, The Netherlands

**Keywords:** thyroid hormone transporters, placental barrier, fetal development, MCT8, OATP1C1

## Abstract

Thyroid hormone is essential for fetal (brain) development. Plasma membrane transporters control the intracellular bioavailability of thyroid hormone. In the past few decades, 15 human thyroid hormone transporters have been identified, and among them, mutations in monocarboxylate transporter (MCT)8 and organic anion transporting peptide (OATP)1C1 are associated with clinical phenotypes. Different animal and human models have been employed to unravel the (patho)-physiological role of thyroid hormone transporters. However, most studies on thyroid hormone transporters focus on postnatal development. This review summarizes the research on the thyroid hormone transporters in pregnancy and fetal development, including their substrate preference, expression and tissue distribution, and physiological and pathophysiological role in thyroid homeostasis and clinical disorders. As the fetus depends on the maternal thyroid hormone supply, especially during the first half of pregnancy, the review also elaborates on thyroid hormone transport across the human placental barrier. Future studies may reveal how the different transporters contribute to thyroid hormone homeostasis in fetal tissues to properly facilitate development. Employing state-of-the-art human models will enable a better understanding of their roles in thyroid hormone homeostasis.

## 1. Introduction

Thyroid hormone is the common name for the precursor thyroxine (T4) and the bioactive hormone 3, 5, 3′-triiodothyronine (T3). Thyroid hormone exerts its genomic action through the binding of T3 to nuclear receptors, which bind to T3-response elements to regulate gene transcription [1]. The intracellular thyroid hormone levels are regulated by metabolizing enzymes, most importantly deiodinases (DIO1, DIO2, and DIO3) [2], and plasma membrane transporters that control cellular influx and efflux of thyroid hormone. DIO2 is the activating deiodinase that converts T4 to T3, and DIO3 is the inactivating one that converts T4 to inactive 3,3′,5′-triiodothyronine (rT3) and T3 to 3,3′-diiodothyronine (T2). DIO1 has both activating and inactivating properties [2]. In addition, sulfotransferases may contribute to thyroid hormone clearance as they catalyze iodothyronines to sulfated iodothyronines (e.g., T3 to T3S and T4 to T4S) which are more efficiently deiodinated by DIO1 [3].

Thyroid hormone is essential for normal fetal development, particularly for the fetal central nervous system (CNS). The fetus depends on the maternal thyroid hormone supply, particularly in the first 16 weeks of gestation when the fetal thyroid gland is not fully functional yet [4]. Several clinical studies have shown that low or high maternal free thyroxine (FT4) concentrations during early pregnancy are negatively associated with neurocognitive function in the offspring [5,6,7]. Before reaching the fetal target cells, thyroid hormone has to be transported across the placental barrier and across the blood–brain barrier (BBB) and/or blood–cerebrospinal fluid barrier (BCSFB) to reach the fetal brain. Thyroid hormone transporters govern the processes of influx and efflux of thyroid hormone across cellular barriers.

Most information on the expression and physiological relevance of thyroid hormone transporters is derived from postnatal models. However, with the recent advancement of Next Generation Sequencing techniques, data has recently also become available on the expression of thyroid hormone transporters in human fetal tissues [8,9]. Roost et al. obtained 21 organs from 17 human fetuses between a gestational week (GW) 8.2 and 22 and generated a collection of human fetal transcriptional profiles at different developmental stages. This work provided valuable expression information on thyroid hormone transporters in human fetal tissues during early and midgestation, which is summarized in Supplementary Figure S1. Thanks to the human cell atlases of gene expression in fetal tissues generated by Cao et al., gene expression in different cell types and tissues can be explored interactively via https://descartes.brotmanbaty.org/ (accessed on 14 September 2022) [8].

This review will summarize studies on substrates, expression and tissue distribution, and physiological and/or clinical relevance of thyroid hormone transporters, with a specific focus on fetal development and the human placental barrier.

## 2. General Overview of Thyroid Hormone Transporters

It was originally believed that thyroid hormone could enter the cells through passive diffusion, given its lipophilic properties and the lipid biolayer of the plasma membrane. In the 1970s, it was demonstrated that thyroid hormone transfer across the plasma membrane is a saturable and energy-dependent process, and the first thyroid hormone transporters were identified later in the 1990s [10]. The substrates of the thyroid hormone transporters have been studied using radiolabeled uptake/efflux assays, mostly in transfected cell lines or cRNA-injected Xenopus oocytes. To date, 15 thyroid hormone transporters from several solute carrier (SLC) families and the adenosine triphosphate binding cassette (ABC) family have been identified, including monocarboxylate transporters (MCT8 and MCT10), L-type amino acid transporters (LAT1 and LAT2), Na^+^/taurocholate co-transporting polypeptide (NTCP), SLC17A4, organic anion transporting peptides (OATP1A2, 1B1, 1B3, 1C1, 2B1, 3A1, 4A1, 4C1) and ABCB1 [11] (Table 1). The SLC thyroid hormone transporters have various transmembrane domains and are classified into different classes according to their polypeptide chain fold patterns: the NhaA fold, the major facilitator superfamily fold, and the LeuT fold. Details of their structures are reviewed in [11,12].

## 3. Thyroid Hormone Transporters during Fetal Development

### 3.1. MCT8

#### 3.1.1. Substrates

MCT8 (SLC16A2) facilitates both the uptake and efflux of T3 and T4 [13,14]. MCT8 also facilitates the uptake of rT3 and 3,3′-T2, but not sulfated T4 or amino acids such as tyrosine, tryptophan, leucine, and phenylalanine [13].

#### 3.1.2. MCT8 Deficiency

Inactivating mutations in MCT8 result in the Allan–Herndon–Dudley syndrome [15]. This disease (also known as MCT8 deficiency) is mostly found in male patients as the *SLC16A2* gene, encoding MCT8, is located on the X-chromosome [15]. Patients with MCT8 deficiency have elevated serum T3 and reduced (free) T4 levels [39]. Typically, they display intellectual disability, hypotonia, poor head control, and are not able to sit or walk independently [39].

Since the murine Mct8 has the same substrate preference as human MCT8, mouse models have been generated to reveal the pathogenic mechanisms of MCT8 deficiency [40]. Adult *Mct8* knockout mice showed elevated T3 and reduced T4 levels in serum, recapitulating the human situation [41,42,43,44]. These mice also showed reduced T3 and T4 content in the brain, which was found in a human fetus with MCT8 deficiency as well [41,42,43,44,45]. Multiple studies have shown the expression of MCT8 in (fetal) cerebral microvessels [16,46,47,48]. Vatine et al. found that induced pluripotent stem cells (iPSC)-derived brain microvascular endothelial cells (iBMECs) from patients with MCT8 deficiency display diminished thyroid hormone transport, particularly for T3 [49]. These studies point to impaired thyroid hormone delivery to the brain.

Strikingly, Ferrara et al. reported higher thyroid-stimulating hormone (TSH) and T4 at embryonic day 18 (E18) and higher serum T4 levels at postnatal day 0 (P0) in serum of *Mct8* knockout mice compared to wild-type (WT) mice [44]. However, T4 became much lower than WT mice in adult mice [41,42,43,44]. In the cerebral cortex, higher T3 and T4 concentrations were found in *Mct8* knockout mice at E18 and P0 compared to WT, whereas reduced T3 and T4 concentrations were present in adult mice [44]. In human neonates, measurements from dry blood samples showed no significant difference in T3 concentrations between MCT8 deficient and healthy neonates [50]. However, MCT8 deficient neonates had significantly lower rT3 and T4 concentrations [39,50], which was also seen in *Mct8* knockout mice [44]. These studies indicated that thyroid function tests in mice versus humans with defective MCT8 might differ across developmental stages.

MCT8 was also found in all layers of the cerebral cortex of the fetal brain, with mRNA expression levels comparable throughout gestation and in adulthood [16,51]. In addition to the BBB, another traditional route of thyroid hormone delivery to the (fetal) brain is via the BCSFB, where MCT8 was present in the epithelium of the choroid plexus and ependymal cells [16,46,47]. Recently two novel routes via the outer and inner CSFBB have been proposed [47]. A detailed study by Lopez-Espindola et al. revealed spatiotemporal differential expression of MCT8 in human fetal brain. MCT8 was present at the outer CSFBB as strong MCT8 protein expression was observed in the leptomeninges and weak expression at the endfeet of radial glial cells from gestational week (GW) 14 onwards [47]. MCT8 was also expressed in the epithelium lining the lateral ventricles, the inner CSFBB, which disappears during later development [47]. To which extent MCT8 transports thyroid hormone to the fetal brain via each of these three routes in vivo is still unclear.

In addition to abnormal thyroid hormone concentrations in serum and brain, all patients with MCT8 deficiency have delayed myelination [39]. Delayed myelination was already observed in a 30-week MCT8-deficient fetus, as evidenced by very low or absent myelin basic protein in the fetal cerebellum [45]. This fetus also displayed delayed maturation of the cerebral cortex and cerebellum and altered synaptogenesis, as evidenced by the absence or little expression of synaptophysin in the frontal cortex and cerebellum [45]. In contrast, *Mct8* knockout mice appeared to have no obvious neurodevelopment deficit or motor abnormalities [41,52], whereas Mct8/Oatp1c1 double knockout mice showed neurological abnormalities [53] (see “OATP1C1” section below for more details). The reason for the difference lies in the strong expression of another T4 transporter, OATP1C1, in the BBB of mice but not of humans [54].

MCT8 is localized in different neuronal populations of various regions of the brain, including the cerebral and cerebellar cortex, hippocampus, striatum, and hypothalamus [17,23,46,55,56]. In mice, despite decreased T3 transport in primary cortical cultures [23,52], Mct8-deficient Purkinje cells did not show differences in dendritic parameters after T3 treatment in vitro [52]. MCT8 deficiency also reduced T3 uptake in human iPSC-derived neuronal cells but did not alter the growth rate and had a similar differentiation profile and T3-dependent neuronal maturation when compared to control cells [49]. However, Mayerl et al. showed impairment in neurogenesis in mice with a conditional *Mct8* knockout in hippocampal neurogenic lineages, indicating cell-autonomous effects of Mct8 deletion in these cells [56].

In addition to the brain, MCT8 mRNA is expressed in various other human fetal tissues (Supplementary Figure S1). However, thyroid hormone entry into the peripheral tissues is not dependent on MCT8 [41], and these tissues of patients with MCT8 deficiency are generally in a thyrotoxic state due to the elevated circulating T3 concentrations. In contrast to *Mct8* knockout mice that showed higher T3 content and DIO1 activity in the liver, a 30-week human fetus with MCT8 deficiency had normal T3 and T4 content in the liver, consistent with the normal T3 concentrations in the neonatal screening [41,42,43,44,45].

#### 3.1.3. (Prenatal) Treatment of MCT8-Deficiency

Multiple therapeutic strategies have been explored for MCT8 deficiency, including using (anti-)thyroid drugs, thyroid hormone analogs, gene therapy, and chemical chaperones [11]. Among them, treatment with thyroid hormone analogs 3,5-diiodothyropropionic acid (DITPA) or 3,3′,5-tri-iodothyroacetic acid (TRIAC) improved the peripheral phenotype [57,58]. As early neurogenesis already starts five weeks after conception, early commencement of treatment may be needed to ameliorate severe neurodevelopmental deficits. Refetoff et al. injected levothyroxine (LT4) via intra-amniotic installations into a fetus with MCT8 deficiency from GW 18 and continued LT4 treatment after birth [59]. This (prenatal) treatment enabled the patient to have near-normal brain myelination at six months and better neuromotor and neurocognitive function when compared to his untreated older brother, who carried the same mutation [59].

Recently, Liao et al. injected adeno-associated virus serotype 9 (AAV9)-Mct8 intravenously into *Mct8/Oatp1c1* double knockout mice, a model that recapitulates the neurological phenotypes of MCT8-deficient patients at a juvenile stage (P30) [60]. AAV9-Mct8 treatment improved locomotor and cognitive performance, although incomplete, in *Mct8/Oatp1c1* double knockout mice compared to the untreated group [60]. Furthermore, the treatment nearly normalized T3 content in various brain regions (thalamus, hippocampus, and parietal cortex) and significantly improved T3-inducible gene expression in the brain and, to a lesser extent, in the liver [60]. In contrast, AAV9-Mct8 treatment had minimal effect on serum thyroid hormone concentrations [60]. The potential of gene therapy was confirmed by a similar study by Sundaram et al. They also showed improved motor coordination, neuronal morphology, and gene expression when AAV-BR1-Mct8, specifically targeting the BBB, was delivered at P0 or P30 intravenously to *Mct8/Oatp1c1* double knockout mice [61].

In addition to gene therapy, Braun et al. used a chemical chaperone, sodium phenylbutyrate (NaPB), in iBMECs carrying a MCT8 pathogenic mutation P321L [62]. NaPB induced the expression of MCT8, either WT or mutant. It also restored T3 and T4 uptake in iBMECs expressing the mutant [62]. In addition, using a pharmacological approach, they identified MCT10 as an alternative transporter contributing to T3 uptake [62].

### 3.2. MCT10

#### 3.2.1. Substrates

MCT10 (SLC16A10) facilitates the influx and efflux of T3, and to a much lesser extent, of T4 [14]. It also transports aromatic amino acids, including phenylalanine, tyrosine, and tryptophan [63,64].

#### 3.2.2. Expression and Tissue Distribution

MCT10 mRNA is expressed in various human fetal tissues, including the liver, kidney, pancreas, placenta, and, to a lesser extent, heart, lung, and stomach (Supplementary Figure S1). In the human fetal brain, MCT10 mRNA is expressed in the cerebral cortex with comparable expression levels throughout gestation and in adults [17]. In addition, weak protein expression of MCT10 was detected in undifferentiated CNS cells from GW and in the microvasculature from GW 10 [16]. MCT10 protein was also detected in the human hypothalamus during the late second trimester and in the apical surface of the choroid plexus [18].

#### 3.2.3. Physiological or Clinical Relevance

*Mct10* knockout mice showed normal T3 and T4 levels in serum and brain, as well as other tissues such as the thyroid, liver, and kidney, and had no obvious neurological defect [43,65]. In *Mct8/Mct10* double knockout mice, thyroid hormone concentrations in the liver, kidneys, and thyroid were even more elevated than in *Mct8* single knockout mice, indicating the contribution of Mct10 to thyroid hormone efflux in these tissues of Mct8 deficiency [43]. Interestingly, *Mct8/Mct10* double knockout mice had normal T4 levels in serum, normalized brain T4 content, and hypothalamic thyrotropin-releasing hormone (TRH) expression, the cause of which is still unclear [43]. Despite expression and localization data in the human fetal brain, the physiological relevance of MCT10 during fetal development has not been investigated.

### 3.3. LATs

#### 3.3.1. Substrates

LAT1 (SLC7A5) and LAT2 (SLC7A8) need the heavy chain CD98/4F2hc (SLC3A2) for their transport activity [66]. Both proteins transport a wide range of L-type amino acids [67]. LAT1 has a preference for 3,3′-T2 but can also transport rT3, T3, and T4 in descending order of efficiency [19]. Apart from uptake, LAT1 also facilitates the efflux of leucine and 3,3′-T2 but not T3 [19].

LAT2 facilitates the uptake of 3,3′-T2 and moderate uptake of T3, but not rT3 or T4 [20,21]. LAT2 does not facilitate the efflux of iodothyronines [20,22].

#### 3.3.2. Expression and Tissue Distribution

LAT1 mRNA is highly expressed in the human fetal brain, liver, placenta, and spleen (Supplementary Figure S1). In the human fetal cerebral cortex, the expression level of LAT1 mRNA is comparable with the adult brain [16]. Lat1 is localized in microvessels in rodents and on the basal surface of the choroid plexus in rats but not in mice [68,69]. Lat1 is also present in a subset of neurons [69].

LAT2 mRNA is highly expressed in the eye, kidney, lung, pancreas, placenta, skin, spleen, and stomach (Supplementary Figure S1). LAT2 mRNA is also detected in the human fetal cerebral cortex with lower levels in GW 17–20 when compared to other gestational ages and adults [16]. In the human adult brain, LAT2 protein is present in neurons, whereas, in the fetal brain, LAT2 is present in microglia but not in neurons [23]. These studies suggest differential spatiotemporal expression of LAT2 in the human (fetal) brain. In mice, Lat2 mRNA is present in the choroid plexus, the hippocampus, the cerebral cortex, and the hypothalamic neurons of the paraventricular nucleus [69].

#### 3.3.3. Physiological or Clinical Relevance

Homozygous global knockout of *Lat1* was embryonic lethal, whereas heterozygous knockout mice are viable, fertile, and phenotypically similar to wide-type littermates with equivalent growth profiles and food intakes [70]. Muscle-specific *Lat1* knockout mice had no overt growth phenotype but showed less efflux of neutral amino acids from the skeletal muscles after fasting [70]. It, therefore, seems likely that the lethality of Lat1 deficiency is mainly due to the reduced transport of neutral amino acids. However, thyroid hormone levels in circulation and tissues were not investigated.

*Lat2* knockout adult mice showed increased urinary loss of small neutral amino acids and slightly impaired movement coordination. The circulating thyroid hormones, TSH, and expression of the thyroid hormone-responsive genes remained unchanged. In addition, no obvious development and growth abnormalities were observed in these mice, and the cerebellar structure appeared unaffected [71]. Another strain of *Lat2* knockout mice only reported decreased T4 and slightly decreased T3 in serum at P21 without hypothyroidism in the liver and brain [71], whereas the *Mct8/Lat2* double knockout had a significant increase of liver DIO1 expression at P0 compared to the *Mct8* single knockout [72]. However, those changes were not observed in other perinatal stages. These observations suggest that LAT2 may contribute little, if any, to the thyroid hormone economy.

### 3.4. NTCP

#### 3.4.1. Substrates

NTCP (SLC10A1) mediates the uptake of (sulfated) bile acids and steroid sulfates such as taurocholate, chenodeoxycholate-3-sulfate, oestrone-3-sulfate, dehydroepiandrosterone sulfate [73,74,75]. It also efficiently facilitates sodium-dependent uptake, but not efflux, of sulfo-conjugated iodothyronines T3S and T4S and marginal uptake of T3 and T4 [24,25].

#### 3.4.2. Expression and Tissue Distribution

NTCP mRNA is exclusively expressed in the hepatocytes in the human liver [11] and is present from GW 9 [9] (Supplementary Figure S1), although significantly lower in the fetal than adult liver [26]. In mice, mRNA expression remains low during fetal development but strongly increases at birth [76]. In mice and rats, Ntcp is localized at the basolateral membrane of differentiated hepatocytes [77], whereas the precise localization of NTCP in the human (fetal) liver has not been studied.

#### 3.4.3. Physiological or Clinical Relevance

*Ntcp* knockout mice showed a hypercholanemia phenotype (including increased serum bile acid (sulfate) concentrations) without obvious abnormalities in the liver, small intestine, large intestine, kidney, pancreas, and spleen, but with thickened gallbladder walls. Pathway analysis of *Ntcp* knockout mice revealed the metabolism of tyrosine, glycine, taurine fatty acid, and glycerophospholipid is significantly dysregulated, as well as the biosynthesis of tryptophan, pantothenate, and CoA [78,79,80]. In humans, the genetic variant Ser267Phe in NTCP caused hypercholanemia in neonates, but their total bile acid levels tended to decrease as age increased [80,81,82,83]. All these studies showed that NTCP deficiency results in changes in bile acids indicating that NTCP is a key player in bile acid transport in vivo. As these studies were not dedicated to investigating thyroid hormone (metabolites), the physiological role of NTCP in thyroid hormone transport in vivo is still unclear.

### 3.5. SLC17A4

#### 3.5.1. Substrates

Until recently, SLC17A4 was known to have some transport capacity towards p-aminohippuric acid and uric acid [84]. This changed when it was identified as a thyroid hormone transporter [28]. SLC17A4 facilitates the uptake of T3 and T4, and to a lesser extent rT3, in a Na^+^ and Cl^−^ independent manner [27]. It also facilitates the efflux of T3 and T4 [27,28].

#### 3.5.2. Expression and Tissue Distribution

SLC17A4 mRNA is expressed in the human fetal intestine and liver from GW 9 [9] (Supplementary Figure S1). In eight-week-old mice, Slc17a4 protein is localized on the apical membrane of the small intestines [84].

#### 3.5.3. Physiological or Clinical Relevance

A recent genome-wide association study (GWAS) showed that variants in the SLC17A4 locus are associated with serum FT4 concentrations, indicating it may be important for thyroid hormone homeostasis (28). To date, no *Slc17a4* knockout animal model has been established. Thus, how and to which extent SLC17A4 affects thyroid hormone homeostasis in (fetal) tissues and during development awaits further investigation.

### 3.6. OATPs

#### 3.6.1. Substrates

OATPs are known as organic anion exchangers, with a broad spectrum of substrates, including sulfobromophthalein sodium, estrone-3- sulfate and dehydroepiandrosterone sulfate. OATPs have overlapping substrates between members, and each member has substrate preference [85]. Friesema et al. first reported that rat Oatp1a1 facilitates the uptake of rT3, T4, T3, and 3,3′-T2 as well as the sulfated iodothyronines in a Na^+^-independent manner [24]. Subsequently, human OATP1A2 was found to transport T3, to a lesser extent, rT3 and T4, as well as their sulfated forms [29,30]. Kullak-Ublick et al. showed that human OATP1B1 and OATP1B3 facilitated the uptake of both T3 and T4 in cRNA-injected Xenopus laevis oocytes in assay buffer with pH 7.5 [31]. Leuthold et al. showed that human OATP2B1 significantly induced uptake of T4 in assay buffer with pH 6.5 but only marginally at pH 8.0 [29,30]. Human OATP1C1 facilitated the uptake of T4, T4S, and rT3 but little uptake of T3 or T3S [32,33]. Both variants of human OATP3A1 facilitated the uptake of T4 but not T3 in cRNA-injected Xenopus laevis oocytes and transfected cells [34,35]. Human OATP4A1 facilitated the uptake of T3 and, to a lesser extent, T4 and rT3 in a Na^+^-independent manner [29], whereas human OATP4C1 facilitated the uptake of T3 and T4 [35,36]. T4 transport by OATPs is stimulated by low extracellular pH with the exception of OATP1C1 [35]. The iodothyronine substrates of human and rodent OATPs are also reviewed in [11,86].

#### 3.6.2. Expression and Tissue Distribution

mRNA of OATP1A2 and OATP1C1 is predominantly expressed in the human (fetal) brain [30,87,88], whereas OATP1B1 and 1B3 mRNA expression is mostly restricted to the liver (Supplementary Figure S1). In the human fetal brain, OATP1C1 is present on both apical and basal-lateral choroid plexus epithelial surfaces from GW 14. OATP1C1 immunoreactivity was also observed in the leptomeningeal cells and blood vessels in the subarachnoid space as well as in ependymocytes and tanycytes, indicating the extensive presence of OATP1C1 at the BBB and the CSFBB [47]. OATP1C1 was detected in radial glial cells in the developing cerebral cortex, in the intermediate zone, subventricular zone, and ventricular zone, with moderate staining in migrating neurons of the raphe nuclear complex [47]. From GW 32, OATP1C1 was detected in astrocytes surrounding the capillary vessels of the blood–brain barrier, but the expression in these vessels was very weak [47]. In rodent adult brain, Oatp1c1 is present in astrocytes, tanycytes, and epithelial cells of the choroid plexus but also at the apical and basal membrane of the endothelial cells of the blood–brain barrier [46,55,89,90,91].

OATP2B1 mRNA is predominantly expressed in the human fetal liver but also in the brain, intestine, lung, placenta, and spleen. OATP2B1 protein is present on the basal-lateral membrane of hepatocytes [30]. mRNA of OATP3A1 is expressed in the human fetal brain, heart, skin, and spleen. Though OATP4A1 mRNA is expressed in many tissues, such as the heart, placenta, lung, liver, skeletal muscle, kidney, and pancreas in human adults [29], in fetal tissues, it is only expressed in amnion and placenta. OATP4C1 mRNA is predominantly expressed in human fetal as well as adult kidneys and liver (Supplementary Figure S1) [11].

Except for OATP1C1, information on the subcellular localization of OATPs in human fetal tissues is still very limited.

#### 3.6.3. Physiological or Clinical Relevance

The Ile13Thr polymorphism in OATP1A2 was associated with higher T3 levels in a population of the Rotterdam Scan Study, whereas Val174Ala was associated with higher T4S levels in healthy blood donors [92,93]. In addition, GWAS studies showed that single-nucleotide polymorphisms (SNPs) of OATP1B1 and OATP1B3 were associated with serum FT4 levels [28,38]. These studies indicate their role in thyroid hormone homeostasis. Most OATPs transport iodothyronines with a relatively low affinity, whereas OATP1C1 exhibits a high affinity for T4 and rT3 [32]. Together with its enriched expression in the human brain, these properties of OATP1C1 suggest its physiological relevance, which was indeed confirmed in mouse models as well as by the clinical phenotype of a patient with OATP1C1 deficiency.

*Oatp1c1* knockout mice showed decreased brain T4 content (~60%) compared to healthy littermates, whereas thyroid hormone in serum and peripheral tissues did not alter [89]. This suggests the importance of Oatp1c1 in T4 transport into the brain in rodents. In addition to decreased T3 and T4 uptake into the brain, *Mct8/Oatp1c1* double knockout mice also showed developmental phenotypes which were not present in *Mct8* single knockout mice, including delayed cerebellar development, reduced myelination, pronounced locomotor abnormalities, and highly compromised differentiation of GABAergic interneurons in the cerebral cortex [53]. As Oatp1c1 is expressed in the mouse BBB, it may compensate for T4 delivery into the brain, which is followed by local conversion to T3 in the brain of *Mct8* single knockout mice [53]. The redundancy of Oatp1c1 as a T4-transporter explains the absence of an overt cerebral phenotype in *Mct8* single knockout mice, suggesting that Oatp1c1 does play a physiologically relevant role for thyroid hormone transport in vivo.

So far, only a single patient with OATP1C1 deficiency has been reported. A girl who harbors a homozygous loss of function mutation Asp252Asn was born normal but manifested with developmental impairments at 10 months of age and later progressed to neurological regression with her brain imaging showing degeneration of grey and white matter and severe glucose hypometabolism. She was also intolerant to colds [94]. With OATP1C1 and DIO2 being co-expressed in radial glia and astrocytes in the human fetal neocortex, the clinical phenotype in this patient is possibly caused by a reduced T4 uptake in OATP1C1-expressing astrocytes, resulting in a reduced DIO2-mediated conversion to T3 [94,95]. The progressive nature of the disease symptoms over time might hint at the role of OATP1C1 in the postnatal period. With the treatment of TRIAC and low-dose LT4, the progression of the clinical course in this patient appeared to be halted or even improved [94].

### 3.7. ABCB1

So far, only a few studies have focused on ABCB1 in relation to thyroid hormone transport. ABCB1 has only been shown to facilitate T3 efflux in over-expressed Madin-Darby canine kidney (MDCK) cells [37]. ABCB1 mRNA is expressed in the human fetal heart, liver, and placenta (Supplementary Figure S1).

Zibara et al. investigated T4 transport across the BSCFB using isolated perfused choroid plexus of the sheep and evaluated the contribution of thyroid hormone transporters using various inhibitors. T4 extraction from blood to choroid plexus decreased by ~34% in the presence of verapamil, a substrate of ABCB1, indicating that ABCB1 contributes to T4 entry into the cerebrospinal fluid [96]. However, caution should be addressed as verapamil is not a specific ABCB1 inhibitor [87]. In humans, a recent genetic analysis study revealed that one SNP of ABCB1 is associated with serum FT4 concentrations [38].

## 4. Thyroid Hormone Transporters in the Human Placental Barrier

During most of the first trimester, the human fetus is surrounded by the amniotic cavity (containing amniotic fluid) and the exocoelomic cavity (containing coelomic fluid), which are separated by a thin membrane. The fetus is bathed in the amniotic fluid, and the secondary yolk sac, an extension of the fetal digestive tract and circulation, is floating in coelomic fluid [97]. The exocoelomic cavity is the site for maternal–fetal molecular exchange and is a physiological liquid extension of the early placenta [97]. From GW 11–12, the secondary yolk sac degenerates and the amniotic cavity grows, making the placenta the site for maternal–fetal nutrients exchange. The early placenta primarily consists of the mono-nucleated cytotrophoblasts, which proliferate and differentiate into the multi-nucleated syncytiotrophoblasts that line the epithelial syncytium, which in turn are in direct contact with maternal blood. From the second half of pregnancy, the cytotrophoblasts, a continuous cell layer beneath syncytiotrophoblasts, start to gradually form sparse columns, leaving the syncytiotrophoblasts as the most important membrane barriers in the maternal–fetal transport process [98].

**Figure 1:**
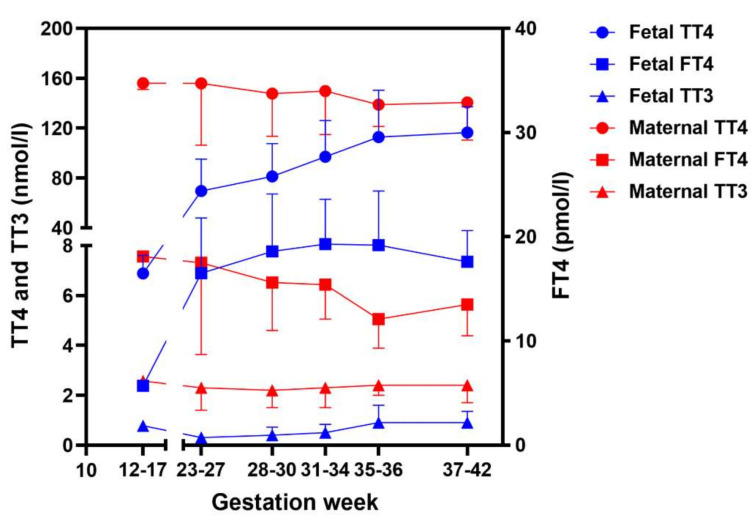
Thyroid hormone levels in maternal and fetal serum during different gestational ages. Data at gestational weeks 12–17, depicted as mean ± SEM, were collected from Calvo et al. [97], which measured concentrations of (free) thyroid hormone and binding proteins in first trimester in amniotic fluids and fetal serum. The other data, depicted as mean ± SD, were from Hume et al. [100], which measured concentrations of (free) thyroid hormone and thyroxine-binding protein during the second and third trimesters in cord blood and the mothers. Note: the data of maternal and fetal FT4 was depicted on the y-axis. TT4: total T4; FT4: free T4; TT3: total T3.

Thyroid hormone was detected in fetal serum, coelomic and amniotic fluid, and brain as early as GW 5–6 when the fetal thyroid gland was not functional yet [97]. Total T4 (TT4) levels in amniotic fluid and coelomic fluid were less than 1% of maternal levels, while FT4 levels were relatively high due to very low thyroxine-binding protein levels. In early pregnancy, TT4 and FT4 levels in fetal serum were lower than in maternal serum, while later in pregnancy, fetal serum levels reached levels similar to maternal circulation (Figure 1). Thyroid hormone has been shown to be transferred across the human placenta by injecting radioactive thyroid hormone into the mothers [99]. Vulsma et al. detected 35–70 nM T4 (25–50% of the normal concentrations) in the cord blood of newborns with fetal thyroid agenesis and dyshormonogenesis [27]. Together, these studies indicate maternal-to-fetal thyroid hormone transfer.

There are several processes that affect the availability of thyroid hormone in the fetus, including thyroid hormone transporters facilitating transcellular transport, intracellular metabolizing enzymes (the deiodinases and the sulfotransferases), and distributor proteins such as transthyretin (TTR, see below).

Though (single cell) RNA sequencing analysis of human placentas revealed the expression of thyroid hormone transporters including LAT1, LAT2, MCT8, MCT10, OATP1A2, OATP4A1, OATP2B1, and ABCB1 [9,88,101] at different stages of pregnancy. mRNA expression of MCT8 and MCT10 increased, whereas ABCB1 decreased in the human placenta in relation to gestational age [88,102]. LAT1 mRNA expression decreased during the first half of pregnancy and then increased to term level by GW 27–34, whereas LAT2 mRNA remained constant throughout pregnancy [88]. Before GW 14 OATP1A2 mRNA was lower than term and increased to term level by GW 27–34, whereas OATP4A1 mRNA had similar levels at GW 6–10 and term, though it was significantly lower at GW 11–14 [88]. In human term placenta, mRNA expression of LAT1, LAT2, and OATP2B1 was relatively high, followed by OATP1A2 and OATP4A1, and MCTs to a lesser extent [87,103].

**Figure 2:**
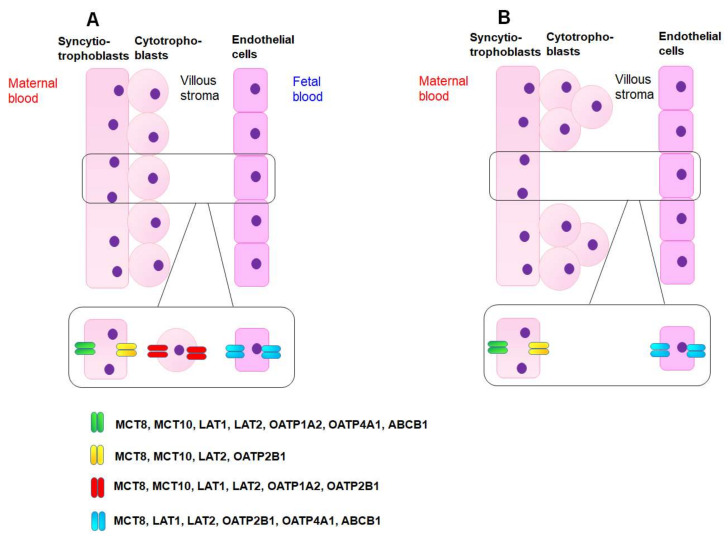
Expression and localization of thyroid hormone transporters in the human placental barrier. A schematic representation of human placental chorionic villi during the first trimester of pregnancy (**A**) and at term (**B**) is depicted. (**A**) During the first trimester, the mononucleated cytotrophoblasts (CTB) proliferate and differentiate into multi-nucleated syncytiotrophoblasts (STB). Both trophoblasts participate in maternal–fetal exchange. As the pregnancy progresses, the CTB gradually forms column structure beneath the STB, leaving the STB primarily controlling maternal–fetal exchange from midgestation. The expression and localization of thyroid hormone transporters are depicted based on the immunostaining studies and single-cell RNA sequencing data mentioned in the text. MCT: monocarboxylate transporter; LAT: L-type amino acid transporter; OATP: organic anion transporting peptide; ABC: adenosine triphosphate binding cassette.

Chan et al. studied the protein expression of all these transporters, except for OATP2B1, in the human placenta [88]. Immunostaining studies showed that MCT8 was localized in both cytotrophoblasts and syncytiotrophoblasts throughout pregnancy, whereas MCT10 was localized in both trophoblasts during the first trimester and in syncytiotrophoblasts at term [88,104]. Weak expression of MCT8 and MCT10 is also present in extravillous trophoblasts and decidual stroma [88,104]. LAT1 was localized predominately at the apical surface of syncytiotrophoblasts [105]. OATP1A2, OATP2B1, OATP4A1, and ABCB1 proteins were present throughout pregnancy and expressed in syncytiotrophoblasts with OATP4A1 preferentially localized on the apical surface and OATP2B1 on the basal surface [88,102,106]. OATP1A2 was also expressed in cytotrophoblasts and extravillous trophoblasts throughout gestation [88]. An overview of the subcellular localization of TH transporters in the placenta is depicted in Figure 2.

The localization of multiple thyroid hormone transporters on the plasma membranes of cytotrophoblasts and syncytiotrophoblasts suggests the redundancy of TH transporters. The contribution of several individual transporters in trans-placental transport was investigated in vitro using human placenta cell lines such as BeWo cells and using the microvillus plasma membrane (MVM) of isolated syncytiotrophoblasts from human term placentas. In BeWo cells, blocking LATs with pharmacological inhibitors led to ~50–60% reduction of T3 uptake, and knockdown of MCT10 with specific siRNA resulted in ~20% reduction suggesting a major role of LATs and MCT10 in T3 transport [87,105]. In contrast, the majority of T4 uptake was not accounted for by the known thyroid hormone transporters indicating the presence of yet unidentified T4 transporters [87]. However, the warrant must be given about these results as the absent expression of MCT8, the most specific thyroid hormone transporter identified so far, in BeWo cells (as well as in other placental cell models, such as JAR and JEG3 cells) constitutes a limitation of such studies using placental cell models. Loubiere et al. confirmed the protein expression of these thyroid hormone transporters in MVM vesicles. Using the MVM of the syncytiotrophoblasts and a pharmacological approach, their study showed that 87% of saturable T3 uptake is contributed by MCTs and 67% of T4 uptake by LATs and MCT10 with a minor role by OATPs [101]. For T4 uptake, it is doubtful, as both LATs and MCT10 are poor T4 transporters [14,19].

Thyroid hormone transport across the human term placenta has also been investigated using an ex vivo placental perfusion system. Mortimer et al. showed that little T4 is transferred to the fetus but that ~8% of maternal T4 was detected in the fetal circulation after 6 h of perfusion when DIO3 activity is blocked by iopanoic acid [107]. This suggests that DIO3, which is abundantly expressed and active in the placenta [108,109], is a major factor controlling thyroid hormone trans-placental transport. Some researchers proposed that transthyretin (TTR), which is secreted by the placenta, is important for trans-placental T4 transport by forming a TTR-T4 complex. This complex enters placental cells via endocytosis and extracytosis, thereby avoiding T4 from inactivation by DIO3. Details regarding deiodinases and transport via the TTR-T4 complex in the human placenta are discussed in the review by Landers et al. [98].

Limited clinical studies have been conducted regarding thyroid hormone transporters and pregnancy outcomes. In pregnancies with intrauterine growth restriction, MCT8 mRNA and protein expression are higher in the early third trimester compared to controls of matched gestational age [104]. The current belief of the pathogenic mechanism of MCT8 deficiency is the defective entry of thyroid hormone into the (fetal) brain. However, the human BBB matures as early as GW 16, indicated by the formation of endothelial tight junction molecules [110], whereas the neurogenesis already starts five weeks after conception. During this critical time window, the fetus is entirely dependent on the maternal supply of thyroid hormone. MCT8 deficient fetuses also have defective MCT8 in the placenta as the placenta is a fetal tissue. It is still unclear whether defective placental MCT8 would restrict thyroid hormone transfer to a fetus with MCT8 deficiency and, thus, the fetal brain, thereby contributing to fetal neurodevelopmental deficits.

## 5. Future Perspectives

### 5.1. Physiological Role of Thyroid Hormone Transporters

The analysis of (single-cell) RNA sequencing techniques aids the cell-type specific expression of transporters. This will inform on the possible roles and redundancies of the different transporters (e.g., MCT8, MCT10, NTCP, SLC17A4, OATP1B1, 1B3, 2B1, and 4C1 are all expressed in hepatocytes). Moreover, the physiological roles of these transporters, if any, in thyroid hormone homeostasis need to be determined. To this end, in addition to the expression and localization, the substrate preference(s) of transporters should also be clear (e.g., MCT10 and LATs are poor T4 transporters and thus are unlikely to contribute to T4 transport in vivo). Clarification of the substrates might be relevant, as it is likely that specific transporters (e.g., MCT8) are more physiologically relevant compared to transporters with a broad substrate range. The transport direction of the transporters should also be further characterized as most studies focused on influx, whereas less attention has been paid to efflux ability.

### 5.2. Models for Placenta and Fetal Tissues

The human placental structure changes across different gestational stages. Different studies indicated changes in transporter expression over time [88,101,104]. Healthy ex vivo human trans-placental models are restricted to term placentas. Early placentas have technical difficulties (e.g., cannulating), precluding large-scale application [107]. Generation of placenta-specific knock-out mice is possible but cumbersome [111]. Obviously, this hampers progress in understanding thyroid hormone transport during early pregnancy.

Animal models have intrinsic limitations in mimicking the human situation, which is particularly relevant for developmental questions. Expression patterns of transporters may differ across species, exemplified by the absence of an overt brain phenotype in *Mct8* knockout mice [43]. Moreover, the neurodevelopmental programs differ between mice and humans, illustrated by the large postnatal component of brain development in mice compared to humans [112]. Therefore, different models might be needed for an appropriate understanding of human processes.

Due to the restricted availability of primary human (fetal) tissues and limitations in current models, as detailed above, new technologies such as human iPSC-derived cells or organoids may be helpful [113]. Often the iPSC-derived models typically resemble fetal rather than adult tissues [114]. Specifically, cerebral organoids and liver organoids have similar gene expression profiles as the fetal neocortex and fetal liver, respectively [115,116,117]. Therefore, such models may be well-positioned to address early developmental questions. Moreover, human iPSC-derived models can be employed for disease modeling and drug testing [44,49,118].

In conclusion, a detailed understanding of transporter specifications and a combination of known and novel models are needed to advance understanding of the role of thyroid hormone transporters in the placenta and fetal tissues.

## Figures and Tables

**Table 1:** Overview of the main characteristics of human thyroid hormone transporters.

Transporter	Gene Name	Substrates Uptake	Substrates Efflux	Expression and Tissue Distribution	Relevance for Thyroid Hormone Physiology	Reference
MCT8	SLC16A2	T3, T4 > rT3 > 3, 3′-T2	T3, T4	Ubiquitously (including brain, eye, liver, kidney, muscle)	MCT8 deficiency (Allan-Herndon-Dudley syndrome)	[9,13,14,15]
MCT10	SLC16A10	T3 >> T4	T3	Ubiquitously (including liver, kidney, pancreas, placenta, heart, lung, stomach, brain)	Unknown	[9,14,16,17,18]
LAT1	SLC7A5	3, 3′-T2 > rT3 > T3 > T4	3, 3′-T2	Brain, liver, placenta, spleen	Unknown	[9,16,19]
LAT2	SLC7A8	3, 3′-T2 > T3	No	Ubiquitously (including eye, kidney, lung, pancreas, placenta, skin, spleen, stomach)	Unknown	[9,20,21,22,23]
NTCP	SLC10A1	T3S, T4S > T3, T4	No	Liver	Unknown	[9,24,25,26]
SLC17A4	SLC17A4	T3, T4 > rT3	T3, T4	Intestine, liver	SNP associated with serum FT4 concentrations	[9,27,28]
OATP1A2	SLCO1A2	T3 > rT3, T4, T3S, rT3S, T4S	Unknown	Brain, gonad	SNPs associated with higher T3 or T4S concentration	[9,29,30]
OATP1B1	SLCO1B1	T3, T4	Unknown	Liver	SNP associated with serum FT4 concentrations	[9,31]
OATP1B3	SLCO1B3	T3, T4	Unknown	Liver	SNP associated with serum FT4 concentrations	[9,31]
OATP1C1	SLCO1C1	T4, T4S, rT3	Unknown	Brain	OATP1C1 deficiency	[9,32,33]
OATP2B1	SLCO2B1	T4	Unknown	Liver, brain, intestine, lung, placenta, spleen	Unknown	[9,29,30]
OATP3A1	SLCO3A1	T4	Unknown	Brain, heart, skin, spleen	Unknown	[9,34,35]
OATP4A1	SLCO4A1	T3 > T4, rT3	Unknown	Amnion, placenta	Unknown	[9,29]
OATP4C1	SLCO4C1	T3, T4	Unknown	Kidney, liver	Unknown	[9,35,36]
ABCB1	ABCB1	Unknown	T3	Heart, liver, placenta	SNP associated with serum FT4 concentrations	[9,37,38]

The (sulfated) iodothyronine substrates of the transporters are listed and > indicates substrate preference. Expression and tissue distribution in human fetal tissues were summarized from Supplementary Figure S1. SNP: single nucleotide polymorphism; FT4: free T4.

## Supplementary Materials

The supporting information can be downloaded at: https://www.mdpi.com/article/10.3390/ijms232315113/s1.
