# Supplementary material for: Thyroid Hormone Transporters in Pregnancy and Fetal Development
Source: Int J Mol Sci. 2022 Dec 1;23(23):15113. doi: 10.3390/ijms232315113 (PMC9737226; doi:10.3390/ijms232315113)
Supplement: Supplementary file 1 [file ijms-23-15113-s001.zip › ijms-2003129-supplementary.pdf]

Thyroid hormone transporters in pregnancy and fetal development

Zhongli Chen, Marcel E. Meima, Robin P. Peeters, W. Edward Visser

Supplementary figure

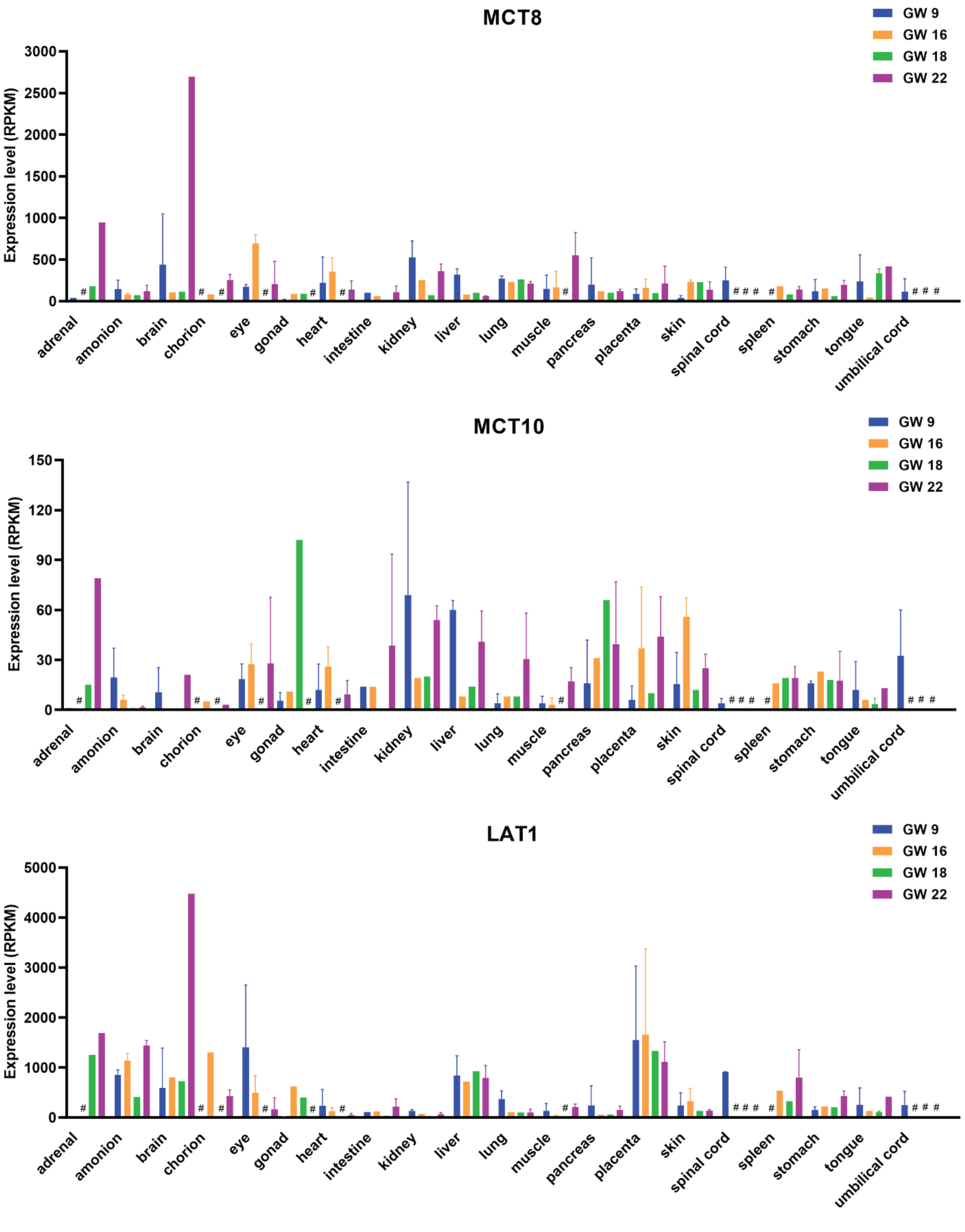
MCT8
MCT10
LAT1
GW 9
GW 16
GW 18
GW 22
Expression level (RPKM)
3000
2500
2000
1500
1000
500
0
150
120
90
60
30
5000
4000
adrenal
amonion
brain
chorion
eye
gonad
heart
intestine
kidney
liver
lung
muscle
pancreas
placenta
skin
spinal cord
spleen
stomach
tongue
umbilical cord
#

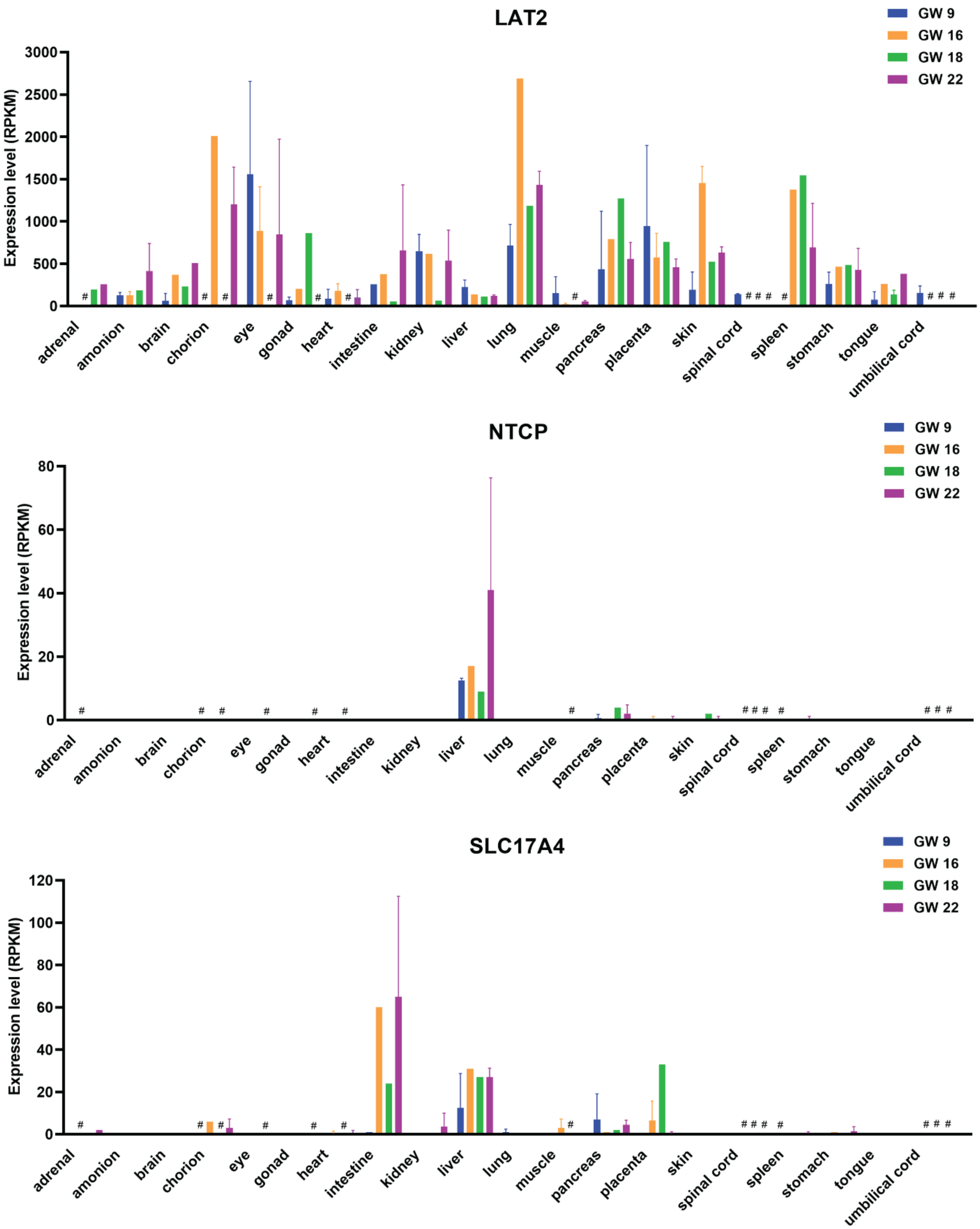
LAT2
GW 9
GW 16
GW 18
GW 22
Expression level (RPKM)
3000
2500
2000
1500
1000
500
0
adrenal
amonion
brain
chorion
eye
gonad
heart
intestine
kidney
liver
lung
muscle
pancreas
placenta
skin
spinal cord
spleen
stomach
tongue
umbilical cord
NTCP
GW 9
GW 16
GW 18
GW 22
Expression level (RPKM)
80
60
40
20
0
adrenal
amonion
brain
chorion
eye
gonad
heart
intestine
kidney
liver
lung
muscle
pancreas
placenta
skin
spinal cord
spleen
stomach
tongue
umbilical cord
SLC17A4
GW 9
GW 16
GW 18
GW 22
Expression level (RPKM)
120
100
80
60
40
20
0
adrenal
amonion
brain
chorion
eye
gonad
heart
intestine
kidney
liver
lung
muscle
pancreas
placenta
skin
spinal cord
spleen
stomach
tongue
umbilical cord

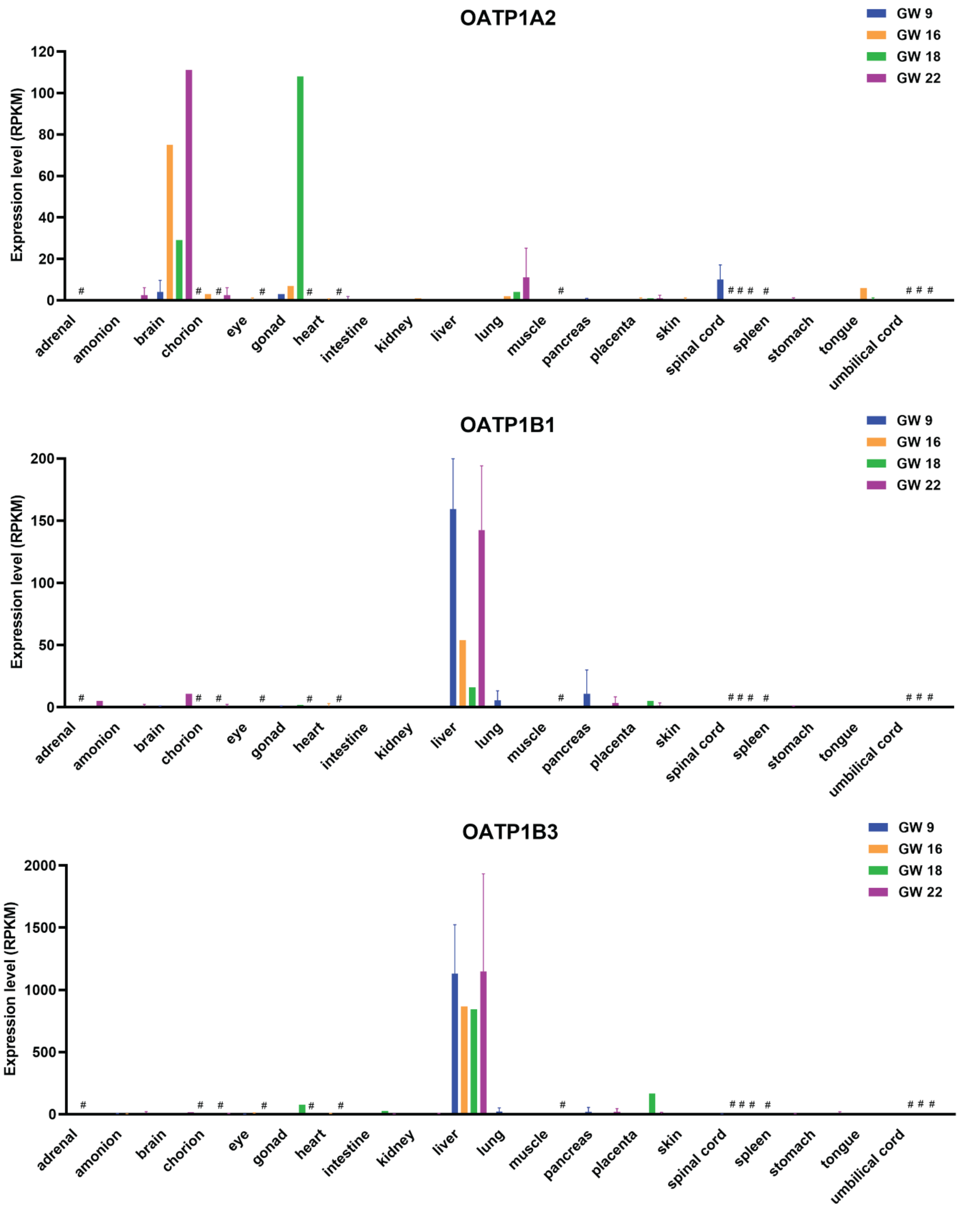
OATP1A2
GW 9
GW 16
GW 18
GW 22
Expression level (RPKM)
0
20
40
60
80
100
120
adrenal
amonion
brain
chorion
eye
gonad
heart
intestine
kidney
liver
lung
muscle
pancreas
placenta
skin
spinal cord
spleen
stomach
tongue
umbilical cord
OATP1B1
0
50
100
150
200
OATP1B3
0
500
1000
1500
2000

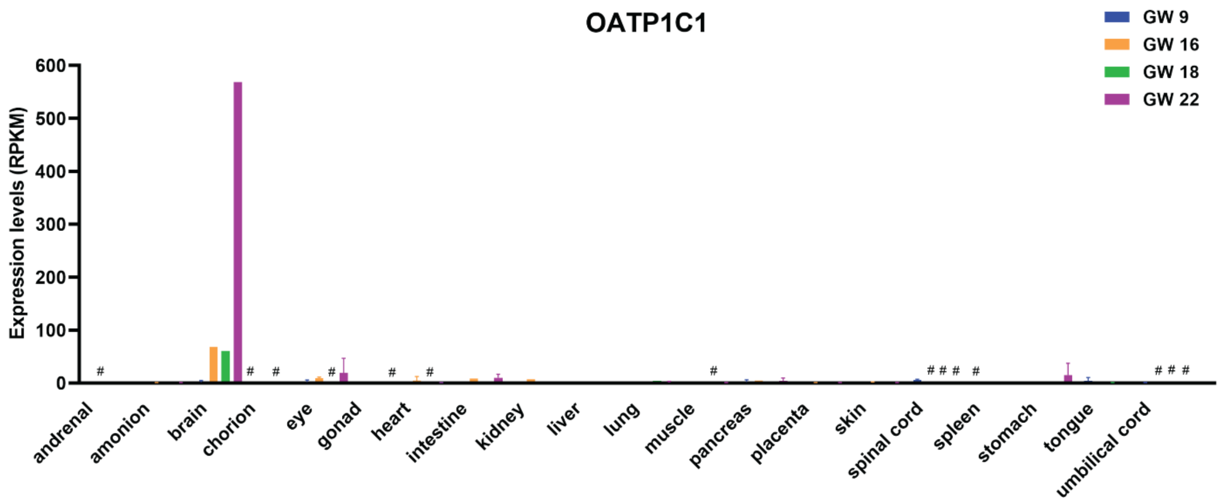
OATP1C1
GW 9
GW 16
GW 18
GW 22
Expression levels (RPKM)
600
500
400
300
200
100
0
andrenal
amonion
brain
chorion
eye
gonad
heart
intestine
kidney
liver
lung
muscle
pancreas
placenta
skin
spinal cord
spleen
stomach
tongue
umbilical cord

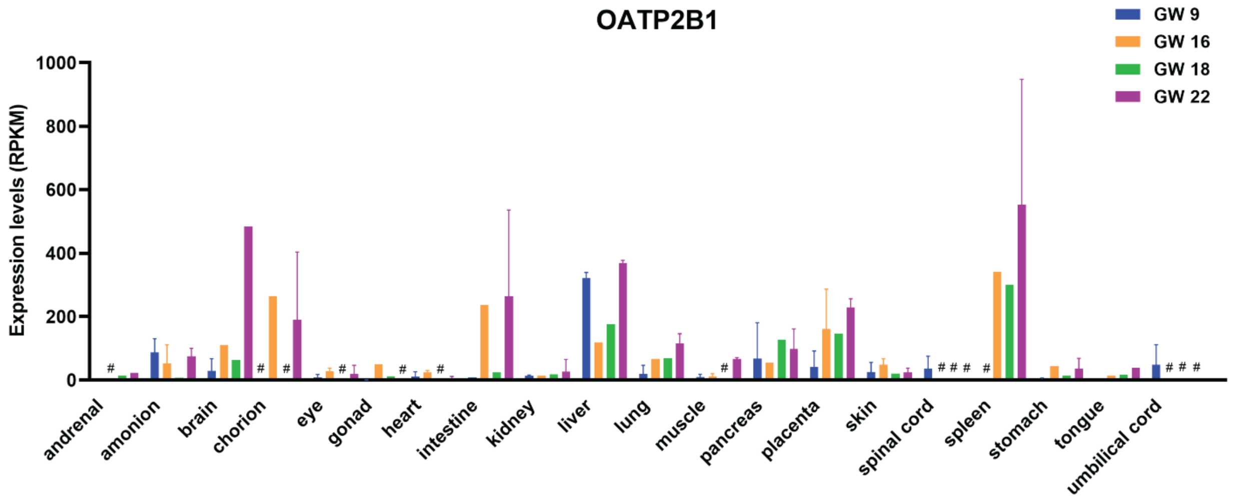
OATP2B1
GW 9
GW 16
GW 18
GW 22
Expression levels (RPKM)
1000
800
600
400
200
0
andrenal
amonion
brain
chorion
eye
gonad
heart
intestine
kidney
liver
lung
muscle
pancreas
placenta
skin
spinal cord
spleen
stomach
tongue
umbilical cord

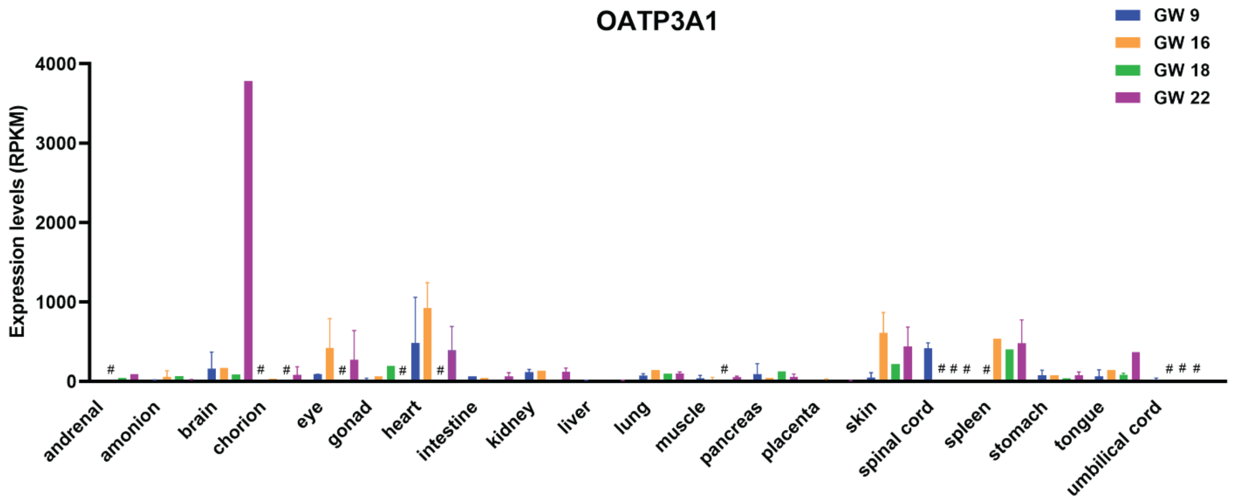
OATP3A1
GW 9
GW 16
GW 18
GW 22
Expression levels (RPKM)
4000
3000
2000
1000
0
andrenal
amonion
brain
chorion
eye
gonad
heart
intestine
kidney
liver
lung
muscle
pancreas
placenta
skin
spinal cord
spleen
stomach
tongue
umbilical cord

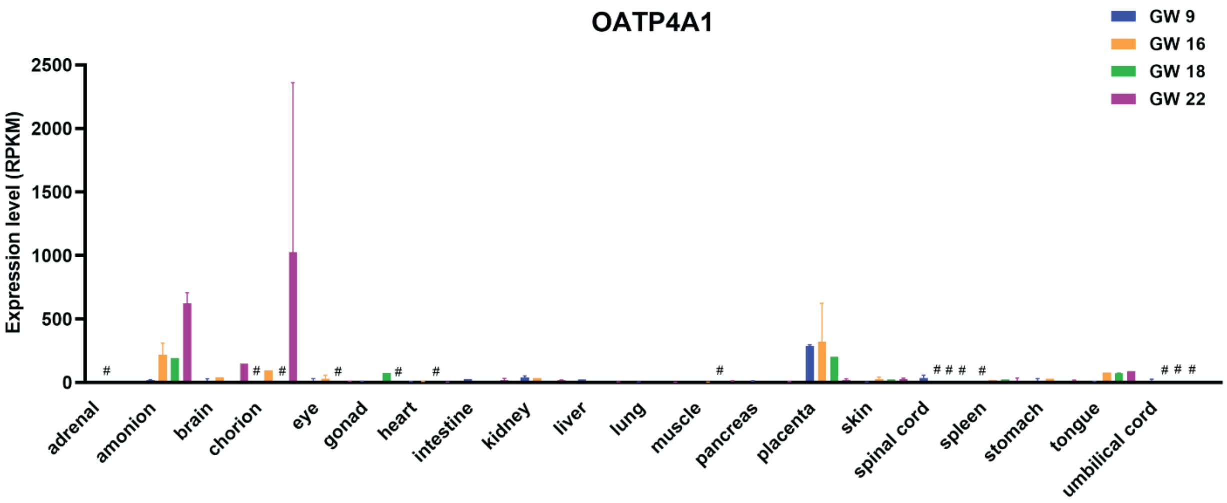
OATP4A1
GW 9
GW 16
GW 18
GW 22
Expression level (RPKM)
2500
2000
1500
1000
500
0
adrenal
amonion
brain
chorion
eye
gonad
heart
intestine
kidney
liver
lung
muscle
pancreas
placenta
skin
spinal cord
spleen
stomach
tongue
umbilical cord

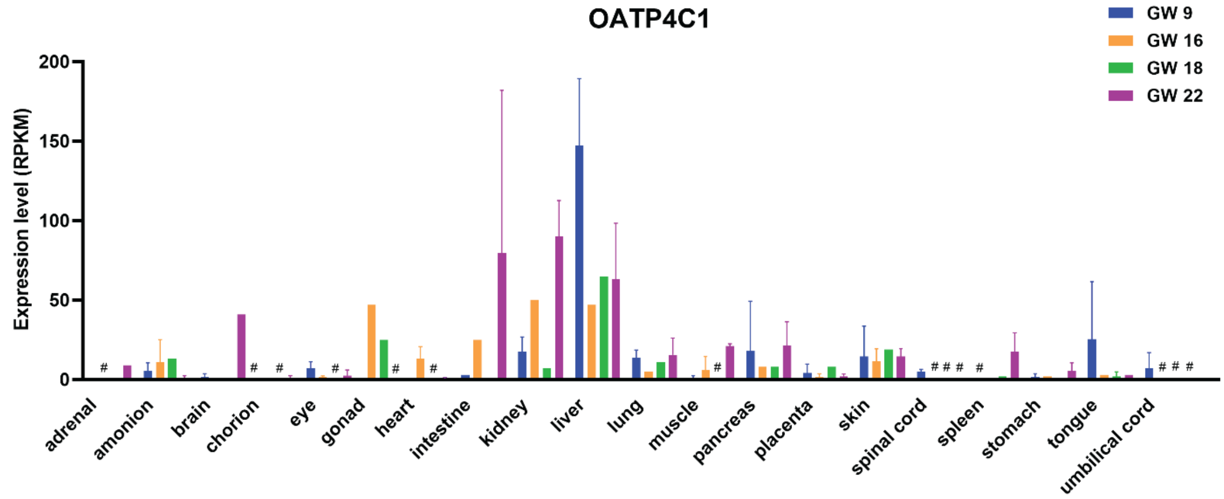
OATP4C1
GW 9
GW 16
GW 18
GW 22
Expression level (RPKM)
200
150
100
50
0
adrenal
amonion
brain
chorion
eye
gonad
heart
intestine
kidney
liver
lung
muscle
pancreas
placenta
skin
spinal cord
spleen
stomach
tongue
umbilical cord

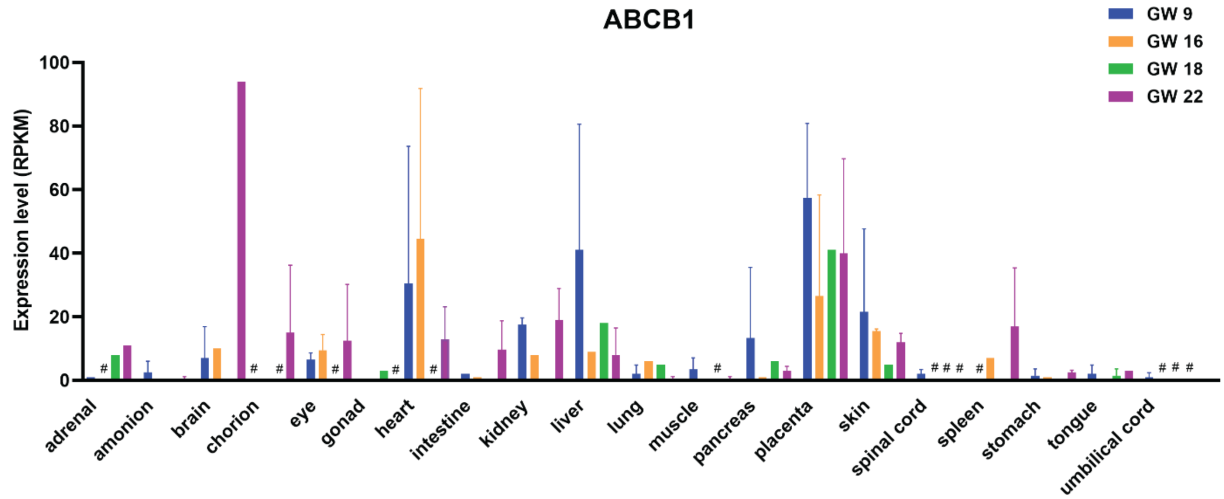
ABCB1
GW 9
GW 16
GW 18
GW 22
Expression level (RPKM)
100
80
60
40
20
0
adrenal
amonion
brain
chorion
eye
gonad
heart
intestine
kidney
liver
lung
muscle
pancreas
placenta
skin
spinal cord
spleen
stomach
tongue
umbilical cord

Supplementary Figure S1. Fetal tissue distributions of thyroid hormone transporters. Expression profiles are based on RNA levels retrieved from (1). Tissues of 20 different human fetal organs, from the first and second trimesters (gestational week (GW) 9, 16, 18 and 22). # indicates no data available for the tissue from a specific gestational week. In case data from two or more tissues from the same gestational week and fetal organ are available, the expression level is depicted as mean ± SD. MCT: monocarboxylate transporter; LAT: L-type amino acid transporter; NTCP: Na+-taurocholate co-transporting polypeptide; SLC: solute carrier; OATP: organic anion transporting peptide; ABC: adenosine triphosphate binding cassette.

1. Roost MS, van Iperen L, Ariyurek Y, Buermans HP, Arindrarto W, Devalla HD, Passier R, Mummery CL, Carlotti F, de Koning EJ, van Zwet EW, Goeman JJ, Chuva de Sousa Lopes SM. KeyGenes, a Tool to Probe Tissue Differentiation Using a Human Fetal Transcriptional Atlas. *Stem Cell Reports*. 2015;4(6):1112-1124.
